# Kinetic Study of Oxidation of Ag-Sn-Zn Solid Solution Powders via Hot Mechanochemical Processing

**DOI:** 10.3390/ma17205115

**Published:** 2024-10-19

**Authors:** Danny Guzmán, Augusto Figueroa, Alvaro Soliz, Alexis Guzmán, Claudio Aguilar, Felipe M. Galleguillos-Madrid, Carlos Portillo, Syed Ismat Shah

**Affiliations:** 1 Departamento de Ingeniería en Metalurgia, Universidad de Atacama, Av. Copayapu 485, Copiapó 1530000, Chile; augusto.figueroa.14@alumnos.uda.cl (A.F.); alvaro.soliz@uda.cl (A.S.); alexis.guzman@uda.cl (A.G.); 2 Departamento de Ingeniería Metalúrgica y de Materiales, Universidad Técnica Federico Santa María, Av. España 1680, Valparaíso 2340000, Chile; claudio.aguilar@usm.cl; 3 Centro de Desarrollo Energético Antofagasta, Universidad de Antofagasta, Av. Universidad de Antofagasta 02800, Antofagasta 1271155, Chile; felipe.galleguillos.madrid@uantof.cl (F.M.G.-M.); carlos.portillo@uantof.cl (C.P.); 4 Department of Materials Science and Engineering and Department of Physics and Astronomy, University of Delaware, Newark, DE 19716, USA; ismat@udel.edu

**Keywords:** electrical contact materials, mechanical alloying, hot mechanochemical processing, kinetic study

## Abstract

Ag-based electrical contact materials are essential in low-voltage devices such as relays, switches, circuit breakers, and contactors. Historically, Ag-CdO composites have been preferred due to their superior electrical and thermal conductivities, resistance to arcing, and mechanical strength. However, the toxicity of Cd has led to increased restrictions on its use. With the aim of contributing to the development of a new environment-friendly, Ag-Zn_2_SnO_4_-based electrical contact material, the kinetics of the hot mechanochemical oxidation of a Ag-Sn-Zn solid solution obtained by mechanical alloying were investigated. The results indicated that the proposed synthesis route produces Ag-based composites with a homogeneous distribution of nanoscale Zn_2_SnO_4_ precipitates, which is unattainable through conventional material processing methods. This kinetic study established that the mechanochemical oxidation of the Ag-Sn-Zn solid solution follows the Johnson–Mehl–Avrami–Kolmogorov model. An analysis of the microstructure and the relationship between the activation energy “*Ea*” and the Avrami exponent “*n*” from experimental data fitting suggests that the primary mechanism for the oxidation of the Ag-Sn-Zn solid solution during the hot mechanochemical process is related to the three-dimensional oxide growth being limited by oxygen diffusion after its immediate initial nucleation.

## 1. Introduction

Ag-based electrical contact materials are fundamental components in various low-voltage devices, including relays, switches, circuit breakers, and contactors. These materials are crucial for establishing and interrupting electrical connections [1]. For an extended period, Ag-CdO composites have been the material of choice for electrical contact applications, primarily due to their outstanding electrical and thermal conductivities, excellent arc erosion resistance, superior resistance to welding adhesion, low contact resistance, high hardness, and suitable mechanical strength [2,3]. Nevertheless, the increasing recognition of the toxic nature of Cd and its compounds [4] has led to growing restrictions on the use of Ag-CdO composites [5].

In this context, Ag-SnO_2_ has become widely recognized as a viable alternative to Ag-CdO for electrical contact materials [6]. However, Ag-SnO_2_ composites exhibit a higher contact resistance than Ag-CdO [7,8]. This behavior is attributed to the formation of a SnO_2_-rich layer on the contact surface during operation, a consequence of the poor wettability between SnO_2_ and molten Ag, as well as the greater thermal stability of SnO_2_ relative to CdO [9,10].

Over the past decade, various metal oxides, including La_2_O_3_ [11,12], Cu_2_O [11], CuO [11], WO_3_ [11], NiO [13], In_2_O_3_ [11,14], TiO_2_ [12], and Bi_2_O_3_ [12], have been incorporated as additives to enhance the mechanical properties of Ag-SnO_2_ and improve the wettability between SnO_2_ and molten Ag. However, the inclusion of non-conductive oxides typically has a detrimental impact on the electrical conductivity of Ag-SnO_2_ composites [15]. In this context, our research group recently reported that it is feasible to mitigate the surface segregation that occurs in Ag-SnO_2_ composites during operation by incorporating ZnO into their structure [16]. In this case, the material was fabricated through hot pressing using Ag, SnO_2_, and ZnO powders. It was verified that the incorporation of ZnO promotes the formation of Zn_2_SnO_4_ on the surface of the material through its reaction with SnO_2_ which is induced by the action of the electric arc according to the following reaction:

SnO_2_ + 2 ZnO → Zn_2_SnO_4_ (1)

Due to the excellent adhesion of Zn_2_SnO_4_ to Ag [17], it has been observed that the formation of this oxide helps limit surface segregation, stabilizing Ag-rich regions within the oxide surface layer. Moreover, the formation of Zn_2_SnO_4_ is associated with a 4% volume contraction, which decreases the overall amount of oxides in the surface layer [16]. Although this strategy for controlling surface segregation proved effective in preventing loss of electrical conductivity of the material during operation, the Ag-SnO_2_-ZnO composite experienced a higher rate of electrical arc erosion compared to its commercial counterpart Ag-CdO.

In this way, it has been demonstrated that the distribution and size of oxide dispersion within the Ag matrix play a crucial role in the densification and subsequent electrical contact behavior of these materials [18,19]. In this context, Zhang et al. [20] investigated the effect of SnO_2_ size on the electrical contact response of a Ag—4 wt% SnO_2_ composite. They reported that reducing the particle size of the oxide phase decreases the duration of the electrical arc and, consequently, the mass loss during operation. Considering this, it is imperative to improve synthesis methods that ensure a fine and homogeneous distribution of oxides within the Ag matrix in order to make the Ag-SnO_2_-ZnO material a viable alternative to replace Ag-CdO composites.

Mechanochemistry is a branch of chemistry that focuses on the chemical and physicochemical transformations of substances in all states of aggregation induced by mechanical energy [21]. It has gained prominence as an efficient and promising method for synthesizing materials that are challenging to obtain through conventional techniques, with applications spanning various fields such as energy and environmental technologies [22], catalysis [23], and pharmaceutical compound synthesis [24], among others.

In recent years, mechanochemistry has garnered significant attention due to its distinctive characteristics, offering substantial benefits over traditional synthetic methods, particularly its ability to initiate chemical reactions without the use of solvents, thereby minimizing the production of toxic byproducts and earning it recognition as a green synthetic process [25]. However, comprehensive control over key reaction parameters in mechanochemical processes, such as reactor temperature and pressure, has yet to be fully established. Currently, most mechanochemical processes are primarily governed by basic variables, such as milling frequency and the weight of the milling media [26].

In this regard, there are only a limited number of studies focused on the synthesis of materials via mechanochemical processes at elevated temperatures. Millet et al. [27] were pioneers in exploring the influence of temperature on mechanochemical synthesis, successfully producing gallium nitride through the hot milling of gallium in a dry ammonia atmosphere using a modified uni-ball mill. More recently, Alić et al. [28] examined the effect of temperature on the mechanochemical synthesis of diamondoid ethers. The milling process was performed using a vibratory mill, with the temperature being controlled by a heating mantle attached to the milling containers. Their findings showed that higher temperatures significantly accelerated the reaction rate, leading to much shorter processing times than conventional solution synthesis methods.

In a previous study, we demonstrated that the oxidation of a Ag-Zn solid solution can be fully achieved through a hot mechanochemical process conducted in an oxygen atmosphere. This research indicated that this novel synthesis method can produce Ag-ZnO composite powders with a fine and homogeneous distribution of nanoscale ZnO precipitates, showcasing a level of control that is unattainable using traditional material processing techniques [29].

Despite efforts, mechanochemistry is still viewed as a “black box” technique. Few works have focused on the kinetic study of mechanochemical transformations, limiting technological transfer from the laboratory to industry. In this context, Gil-González [30,31] proposed a methodology to study the kinetics of non-thermally induced mechanochemical reactions. Based on Butyagin’s work [32], they assumed that the temperature term in the Arrhenius expression could be replaced by the rate of supplied energy in mechanically activated processes. Using the proposed methodology, which correlates milling parameters with the chemical reaction dynamics, it is possible to describe and predict mechanically induced reactions with a high degree of accuracy. Although the work of Gil-González et al. [30,31] represents a significant advancement in the understanding of mechanochemical process kinetics, the proposed methodology does not account for reactions activated by both mechanical and thermal energy.

Given the promising performance of the Ag-ZnO-SnO_2_ composite as an electrical contact material and the proven effectiveness of the hot mechanochemical process in achieving a fine and uniform oxide distribution within the Ag matrix, this study aims to investigate the kinetics of the hot mechanochemical oxidation of a Ag-Sn-Zn solid solution. The principal technological goal was to produce Ag-based powders with a fine and homogeneous oxide distribution, suitable for developing next-generation Ag-based materials for electrical contact applications.

## 2. Materials and Methods

### 2.1. Mechanical Alloying/Ag-Sn-Zn Solid Solution Formation

The mechanical alloying process of 5.28 g Ag (99.9% purity, particle size ≤ 10 µm, Sigma Aldrich, Saint Louis, MO, USA), 0.36 g Zn (>98% purity, particle size ≤ 10 µm, Sigma Aldrich, Saint Louis, MO, USA), and 0.36 g Sn powders (99.5% purity, particle size ≤ 150 µm, Sigma Aldrich, Saint Louis, MO, USA) was carried out in an E-max mill (Retsch GmbH, Haan, Germany) using a rotation speed of 1500 r/min. The E-max mill is a device recently developed by Retsch that combines high impact frequency, intensive friction, and a controlled container circular movement, resulting in a faster size reduction compared to conventional mills [33].

The milling durations were 10, 15, 30, 60, 90, and 120 min. To prevent agglomeration during the process, 0.06 g of stearic acid (Sigma Aldrich, Saint Louis, MO, USA) was added. The milling operations were conducted in an Ar atmosphere to avoid the oxidation of the powders (99.998% purity, AGA Chile, Santiago, Chile). A ball-to-powder mass ratio of 20:1 was used. The milling process was performed intermittently, consisting of 15 min of milling followed by 15 min of rest, with the objective of preventing excessive heating of the vials.

### 2.2. Hot Mechanochemical Processing

The mechanically alloyed powders were subjected to hot milling in a modified attritor (refer to [29] for further details) for 60, 120, 180, 240, 300, and 360 min at temperatures of 25, 50, and 75 °C under an air atmosphere. Higher temperatures were not tested in order to prevent the recrystallization of the powders. This approach avoids an excessive increase in ductility and reduces the probability of agglomeration. The internal temperature was recorded before and after each experiment. The milling chamber was loaded with 14 g of powder and 1400 g of milling balls. Based on previous experiences from our research group regarding the hot reactive milling process in the Ag-Zn system [29], the impeller rotation speed was consistently maintained at 400 r/min.

### 2.3. Characterization

**Figure 1:**
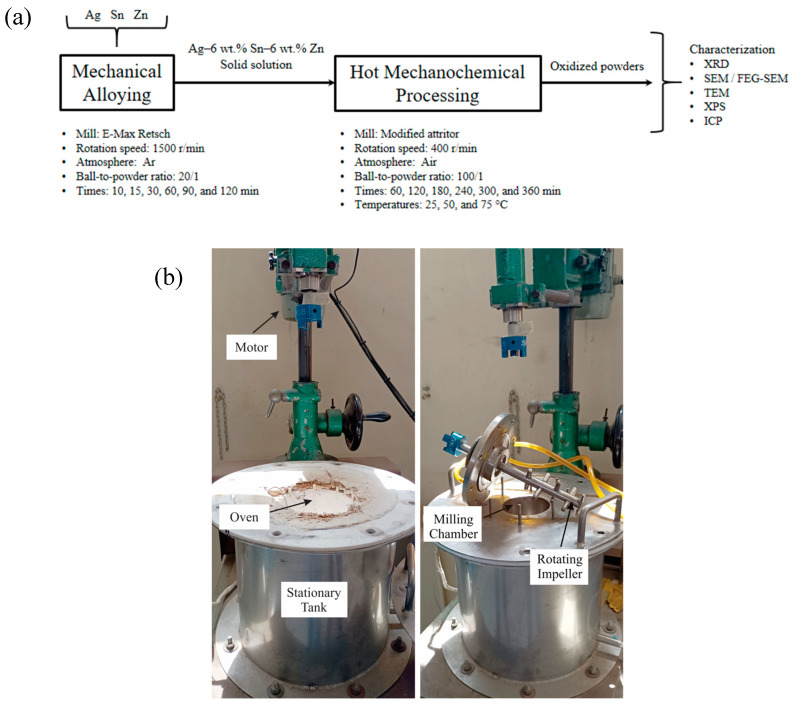
(**a**) Schematic flowchart of the experimental methodology and (**b**) photographs of the modified attritor.

The phase evolution during mechanical alloying and hot mechanochemical processing was examined using X-ray diffraction (XRD) and scanning electron microscopy (SEM). XRD analyses were conducted on a Shimadzu XRD-6000 diffractometer (Kyoto, Japan) with an angular step size of 0.02° (2θ), utilizing Cu Kα radiation and a counting time of 4 s per step. The XRD patterns were interpreted using the Rietveld method [34] through the Material Analysis Using Diffraction (MAUD version 2.999) software [35]. The morphology of the milled powders was assessed with a Zeiss EVO MA10 thermionic microscope (Oberkochen, Germany), and cross-sectioned samples were analyzed using a Zeiss Sigma 500 VP field emission gun SEM (FEG-SEM, Oberkochen, Germany). Transmission electron microscopy (TEM) was performed on a JEM-2010F, 200 kV Field Emission machine (JEOL Ltd., Tokyo, Japan) equipped with an EDAX detector. Furthermore, some mechanochemically processed samples were evaluated using X-ray photoelectron spectroscopy (XPS) with a Thermo Scientific K-Alpha XPS system featuring an Al-Kα monochromatic X-ray source (Thermo Fisher Scientific Instruments, Waltham, MA, USA). Finally, Fe contamination during mechanical alloying was quantified via inductively coupled plasma spectrometry (ICP) using a Perkin Elmer Optima 8000 instrument (Waltham, MA, USA). Figure 1 presents a schematic flowchart of the experimental methodology and photographs of the experimental setup.

## 3. Results and Discussion

### 3.1. Mechanical Alloying/Ag-Zn-Sn Solid Solution Formation

**Figure 2:**
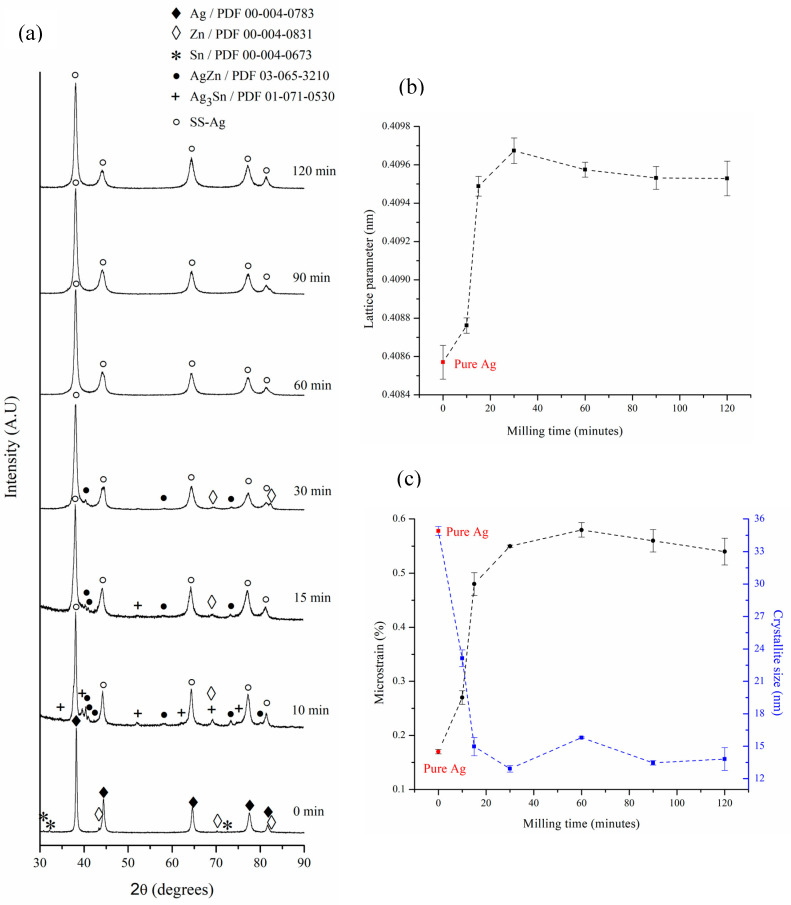
(**a**) XRD patterns of the powder at different mechanical alloying times; (**b**) lattice parameter; and (**c**) microstrain and crystallite size of SS-Ag (Ag-based solid solution) as a function of milling time.

Figure 2a presents the XRD patterns of the samples produced at different milling times. To monitor the phase evolution throughout the mechanical alloying process, the XRD patterns were normalized to the point of maximum intensity. During the first 10 min of milling, detecting the remaining Zn is possible. Additionally, the appearance of the AgZn (ζ) and Ag_3_Sn (ε) phases, as a result of the mechanical energy applied to the powders, is observed. The AgZn (ζ) phase is a stable Ag-Zn solid solution; it has an HCP structure, and its composition is between 26–38.8 wt% Zn. The Ag_3_Sn (ε) phase is an orthorhombic intermetallic in the Ag-Sn system, which is stable in the composition range between 25.5 and 27 wt% Sn. In this range, the Ag lattice parameter increases from 0.40857 ± 8.8 10^−5^ to 0.40876 ± 4.0 10^−5^ nm (Figure 2b), which is evidence of Ag solid solution (SS-Ag) formation. Between 10 and 15 min, a reduction in the diffraction intensities of the Ag_3_Sn (ε) intermetallic is observed. This reduction coincides with the increase in the lattice parameter of SS-Ag shown in Figure 2b in this same time range. Considering that the effective atomic volume of Sn (27.65 Å^3^) is greater than that of Ag (17.06 Å^3^) [36], it can be concluded that at the beginning of the mechanical alloying process in the Ag—6 wt% Sn—6 wt% Zn system, a preferential incorporation of Sn atoms is produced inside the Ag structure, which leads to its expansion.

Between 30 and 60 min of milling, the disappearance of the remaining Zn and AgZn (ζ) solid solution is observed. From this time onward, only a SS-Ag can be detected. This observation can be corroborated by analyzing Figure 2b, where it can be seen that between 30 and 60 min, the lattice parameter of SS-Ag experiences a reduction from 0.40967 ± 6.7 10^−5^ to 0.40957 ± 3.9 10^−5^ nm, remaining practically constant for milling times greater than 60 min (0.40954 ± 6.3 10^−5^ nm, on average). Considering the lower effective atomic volume of Zn (15.24 Å^3^) [36] in comparison with Ag and Sn, the reduction in the lattice parameter of SS-Ag between 30 and 60 min of milling would be related to the preferential entry of Zn atoms into its structure due to destabilization of the remaining Zn and AgZn (ζ) solid solution in this time range.

It can be concluded that a Ag—6 wt% Sn—6 wt% Zn solid solution can be successfully achieved after 60 min of mechanical alloying under the specified experimental conditions. The proposed phase transformation sequence is as follows:

Ag + Zn + Sn → SS-Ag + Zn + Ag_3_Sn (ε) + AgZn (ζ) → SS-Ag + Zn + AgZn (ζ) → SS-Ag (2)

Figure 2c shows the variation in the crystallite size and microstrain of SS-Ag as a function of milling time. Popa’s model [37] was utilized for embedding the anisotropy effect. It can be observed that the crystallite size rapidly reduces from 34.9 ± 0.4 to 15.0 ± 0.8 nm during the first 15 min of milling. After that, the crystallite size does not experience any changes, appearing to have reached a minimum value. The decrease in crystallite size is accompanied by a corresponding increase in microstrain as milling time progresses. During the first 30 min of mechanical alloying, the microstrain rises sharply from 0.17 ± 0.004 to 0.55 ± 0.002%, driven by the high density of structural defects introduced in the initial milling stage. Subsequently, the microstrain stabilizes at an average value of 0.56 ± 0.020%. The minimum crystallite size in the mechanical alloying processes is determined by the competition between the plastic deformation and dislocation motion that tends to decrease the grain size, as well as the recovery and recrystallization behaviors of the material that tend to increase the grain size [38].

**Figure 3:**
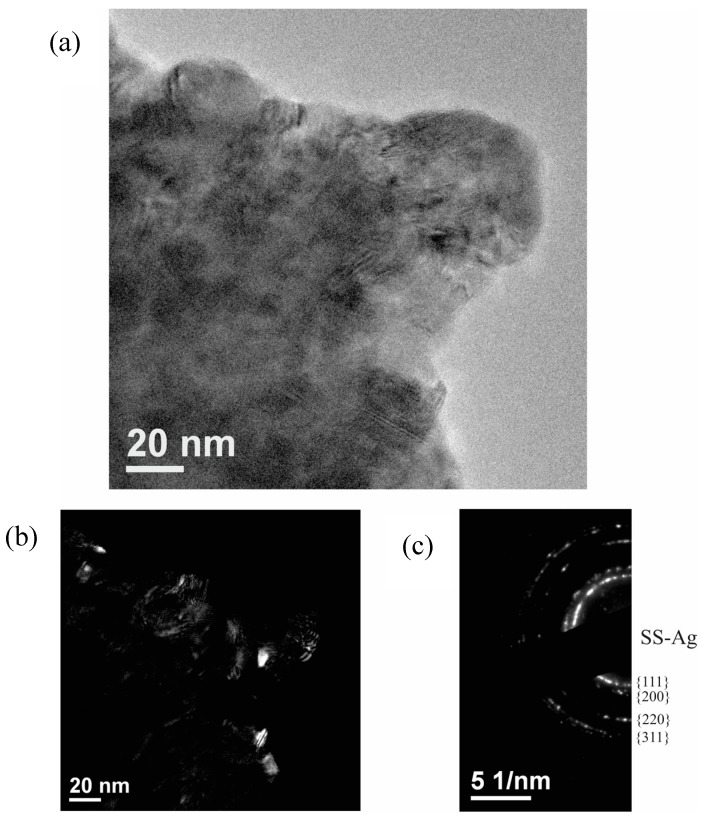
(**a**) TEM image of powders milled for 60 min, (**b**) dark-field image, and (**c**) corresponding electron diffraction pattern.

A TEM analysis was conducted to confirm the nanocrystalline structure of SS-Ag. Figure 3a shows a TEM image of the powders after 60 min of milling. Figure 3b provides a dark-field image with the corresponding electron diffraction pattern. Based on these results, it can be confirmed that the sample consists solely of nanocrystalline SS-Ag with an average crystallite size below 20 nm. No other phases were detected in the electron diffraction pattern, corroborating the XRD findings.

To quantify the extent of Fe contamination, samples subjected to 30, 60, 90, and 120 min of mechanical alloying were analyzed using inductively coupled plasma spectrometry. The results indicated that Fe contamination was below 0.025 wt% in all analyzed samples. This confirms that the variation in the lattice parameter of SS-Ag was primarily due to the incorporation of Zn and Sn atoms into the Ag lattice rather than Fe contamination.

**Figure 4:**
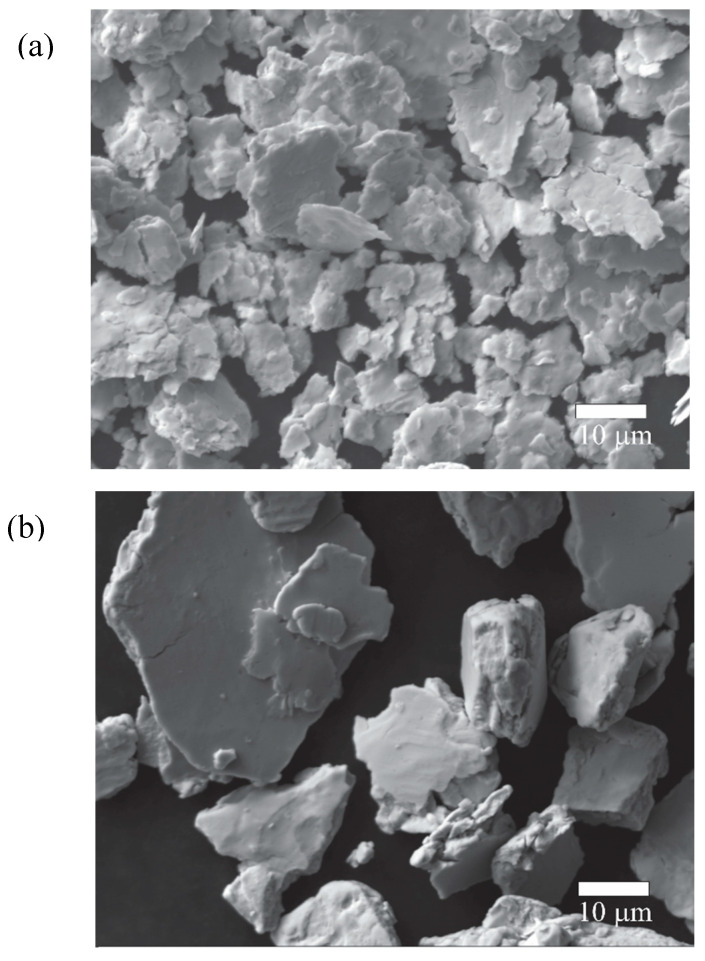
Morphology of the powders after (**a**) 30 and (**b**) 120 min of milling.

Figure 4 illustrates the evolution of powder morphology throughout the mechanical alloying process. In the early stage of milling (Figure 4a), the powders consist of flattened agglomerates resulting from the ductile nature of Ag, Zn, and Sn. These metals undergo plastic deformation and cold welding due to the high energy imparted to the powder. At this stage, the agglomerates have an average diameter of 11.77 ± 2.18 µm, which is larger than the initial Ag powder particles. As the milling process progresses, the flat agglomerates undergo fracture due to the high levels of plastic deformation accumulated during milling. After 120 min of milling, the flattened particles cold weld together, forming larger, rounded agglomerates (Figure 4b) with an average particle size of 32.78 ± 8.04 µm.

As mentioned previously, it can be concluded that it is feasible to produce Ag—6 wt% Sn—6 wt% Zn solid solution powders through mechanical alloying under the evaluated conditions. Given the microstructural and morphological characteristics of the powders obtained, in addition to their potential application in the synthesis of electrical contact materials, they could also be considered raw materials for the fabrication of high-melting-point solder alloys. Such alloys are particularly suitable for high-power energy conversion systems, which operate at elevated temperatures where traditional Sn-based solder alloys become impractical [39]. In this context, Huang et al. [40] studied the production of Ag solid solution powders with 10 wt% Sn via mechanical alloying and evaluated the potential use of these powders as high-melting-point solder alloys. They reported that the incorporation of Sn plays a crucial role in enhancing the degree of sintering in the composite material, promoting a bond with low porosity, which inhibits oxygen penetration and reduces the probability of oxidation. Additionally, it was established that the presence of Sn limits grain growth in the solid solution, improving the mechanical stability of the solder material. Based on these results, it is proposed that Ag-Sn-Zn solid solution powders synthesized through mechanical alloying could have significant potential as high-temperature solder powders, as both Sn and Zn in solution may retard grain growth during sintering. Furthermore, due to the severe plastic deformation induced during milling, the high density of crystalline defects in the powders would facilitate diffusion processes, promoting the sintering of the particles.

### 3.2. Hot Mechanochemical Processing

**Figure 5:**
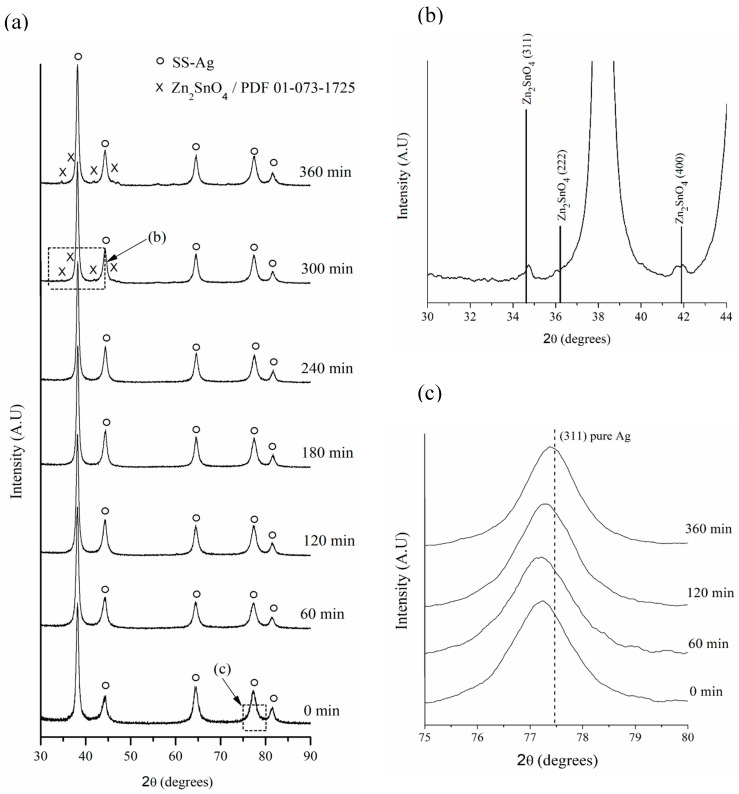
(**a**) XRD patterns of milled powders at 75 °C. Magnified views of (**b**) the XRD pattern of milled powders after 300 min, and (**c**) the XRD patterns of the Ag (311) plane at different milling times.

Figure 5a shows the powder XRD patterns of mechanically alloyed powders after 0, 60, 120, 180, 240, 300, and 360 min of hot mechanochemical processing at 75 °C. As can be seen in Figure 5b, which presents a broad area of the diffractogram of the powders milled for 300 min, it is possible to detect the presence of Zn_2_SnO_4_ by X-ray diffraction from 300 min of processing onward.

It is important to note that under the tested conditions, it was not possible to determine the mechanism of the formation of Zn_2_SnO_4_ induced by the hot mechanochemical process. However, based on the difference in free energy of formation between Zn_2_SnO_4_, SnO_2_, and ZnO [41], it is expected that the mechanochemical process will initially produce the appearance of SnO_2_ and ZnO, followed by the formation of Zn_2_SnO_4_ through the reaction of these elemental oxides.

**Figure 6:**
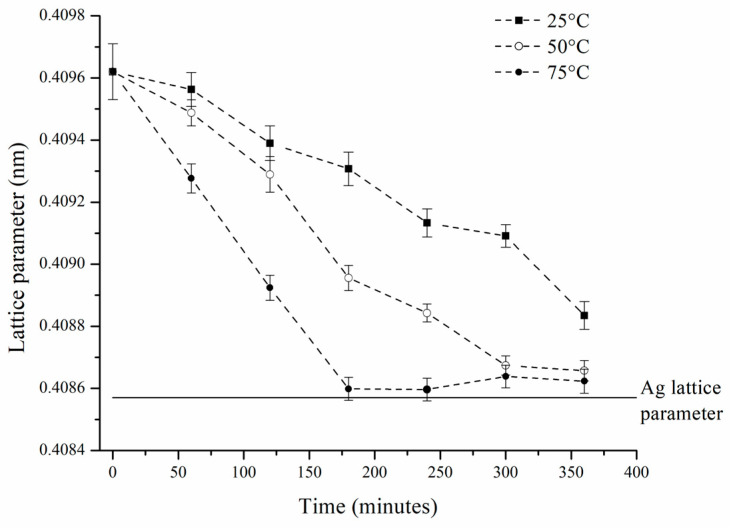
Lattice parameters of SS-Ag as a function of hot mechanochemical processing time for the three temperatures employed.

Additionally, it was observed that the peaks corresponding to SS-Ag gradually shifted toward higher 2θ angles (Figure 5c). This shift is attributed to the reduction in the lattice parameter of SS-Ag, caused by the loss of solute elements (Sn and Zn) due to oxidation during the hot mechanochemical process. The lattice parameter reduction continued until it approached a value close to that of pure Ag (Figure 6). It is important to highlight that all three sample sets processed at different temperatures exhibited similar behavior. However, it was evident that the rate of lattice parameter reduction increased with rising milling temperatures, suggesting that, as expected, the oxidation process is thermally activated when mechanically assisted.

**Figure 7:**
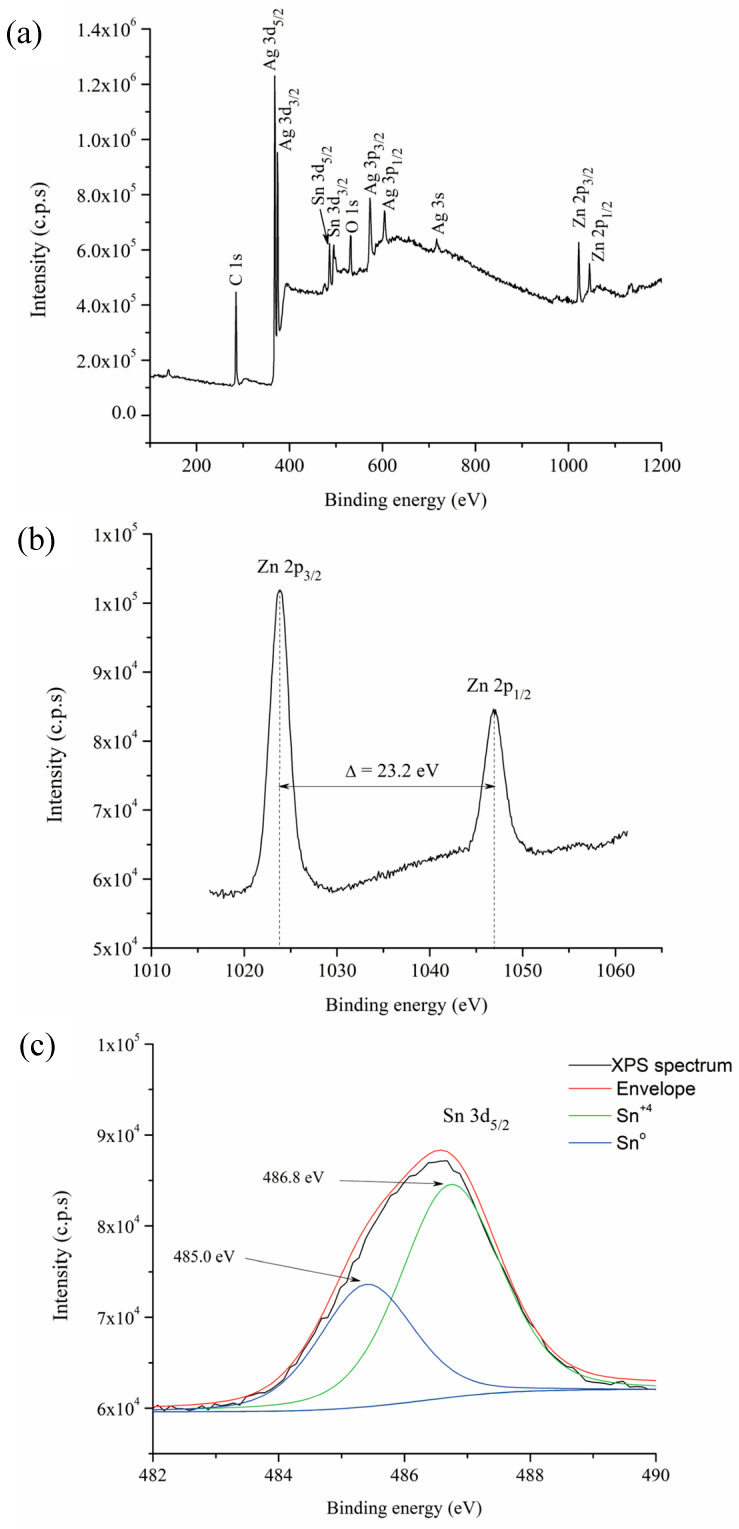
XPS spectra of powders milled for 360 min at 75 °C: (**a**) XPS survey spectrum, (**b**) Zn 2p spectrum, and (**c**) Sn 3d spectrum.

To confirm the oxidation of Sn and Zn from the Ag-based solid solution during the hot mechanochemical process, a sample subjected to 360 min of milling at 75 °C was analyzed using XPS. All binding energies in the XPS analysis were adjusted by referencing the C 1s line to 284.6 eV. Figure 7a shows the full XPS survey spectrum, where the peaks corresponding to the Ag, Zn, Sn, O, and C elements are visible. The presence of C is likely due to organic residues from the stearic acid, which was used as a process control agent during mechanical alloying.

Figure 7b,c present the high-resolution spectra for the Zn 2p and Sn 3d regions, respectively. The Zn 2p spectrum exhibits a doublet with binding energies of 1023.4 eV and 1046.5 eV, corresponding to the Zn 2p_3/2_ and Zn 2p_1/2_ components. The observed energy separation between these two peaks, 23.2 eV, confirms that the majority of Zn is in the +2 oxidation state [42], which is consistent with the presence of Zn_2_SnO_4_ [43].

On the other hand, the Sn 3d_5/2_ peak exhibits an asymmetry that can be deconvoluted into two separate contributions (Figure 7c). The first component, observed at 486.8 eV, corresponds to Sn in the +4 oxidation states, which is consistent with the presence of Zn_2_SnO_4_ [43]. The second peak, located at 485.0 eV, indicates the presence of metallic Sn in the milled powders [44].

XPS results indicate that oxidation of both Zn and Sn occurred during the hot mechanochemical process of the Ag-based solid solution, corroborating the XRD findings which detected the presence of Zn_2_SnO_4_. The presence of elemental Sn and Zn oxides cannot be ruled out, as their size and quantity may be below the detection limits of X-ray diffraction. Additionally, the results suggest that a small proportion of unoxidized Sn may remain within the Ag-based solid solution. This can be attributed to the lower change in the Gibbs free energy during the formation of SnO_2_ compared to ZnO [41].

**Figure 8:**
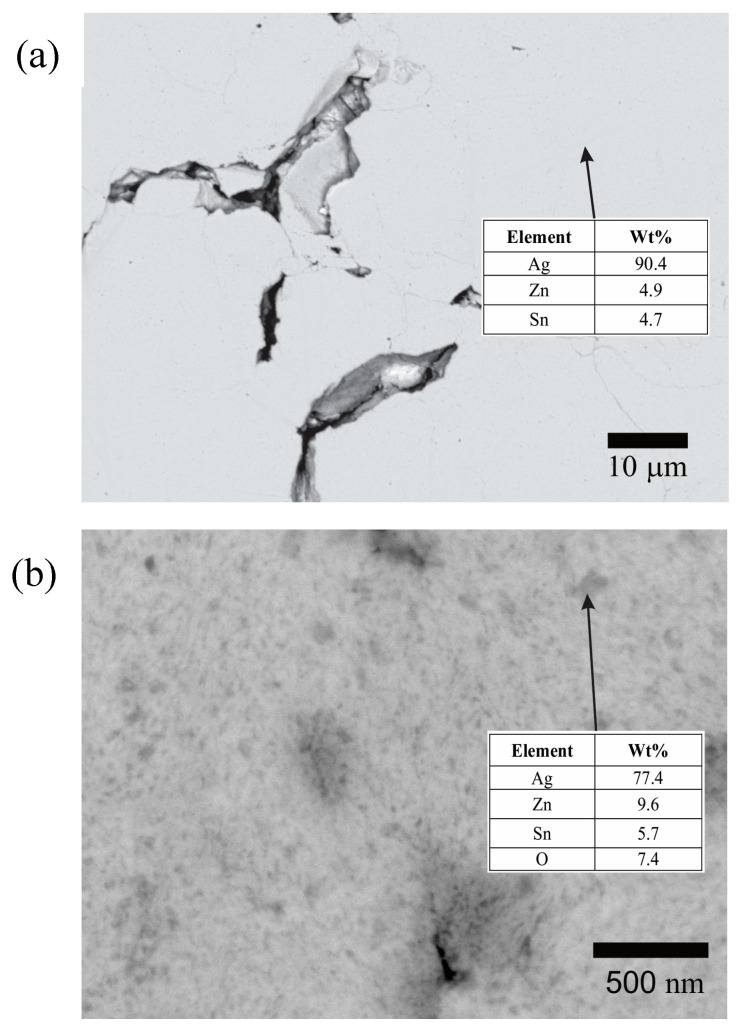
Backscattered electron images of cross-sectioned powder obtained after (**a**) 120 min of mechanical alloying and (**b**) 360 min of hot mechanochemical processing at 75 °C. The tables present the energy-dispersive spectroscopy analyses of selected areas.

In order to determine the change in the phase distribution during the hot mechanochemical processing, powders mechanically alloyed for 120 min and mechanochemically processed for 360 min at 75 °C were metallographically prepared and observed by FEG-SEM in the backscattered electron mode (Figure 8).

The cross-sectional micrograph of the powders mechanically alloyed for 120 min (Figure 8a) reveals a homogeneous microstructure. The Zn and Sn contents in this sample are close to the nominal values of the initial powder mixture (Ag—6 wt% Sn—6 wt% Zn). The slight loss of Zn and Sn detected through the EDS analysis can be explained by the mechanical alloying process, during which the ball and vial become coated with a thin layer of the milled material. This layer may be richer in Zn and Sn than Ag, likely due to the stronger chemical interaction between these elements and Fe. These findings corroborate the XRD analysis results, indicating that the powders mechanically alloyed for 120 min consist of a homogeneous solid solution of Ag, Sn, and Zn.

On the other hand, the cross-section micrograph from the powders mechanochemically processed for 360 min at 75 °C reveals the presence of oxide precipitates on a nanometric scale (dark gray points) uniformly distributed in the Ag-rich matrix (light gray zone) (Figure 8b). The EDS analysis of the dark gray points indicates a higher Zn, Sn, and O content than the solid solution produced by mechanical alloying. This corroborates the fact that the hot mechanochemical process promotes the preferential oxidation of Zn and Sn.

**Figure 9:**
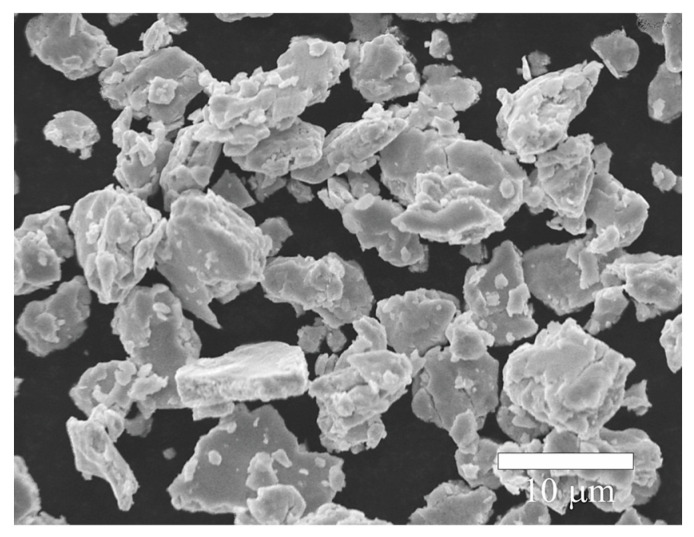
Morphology of the powders after 360 min of hot mechanochemical processing at 75 °C.

Regarding the particle size of the powders processed by hot mechanochemical processing, it was found that all samples exhibited particle sizes below 25 µm. As a representative example, Figure 9 shows the morphology of the powders after 360 min of hot mechanochemical processing at 75 °C. It can be observed that the particles are composed of small, irregular agglomerates with an average diameter of 7.5 ± 3.5 µm. The reduction in particle size compared to powders subjected to mechanical alloying can be explained by the formation of oxide precipitates during the hot reactive milling process, which increases the fragility of the powders and facilitates their fracture.

**Figure 10:**
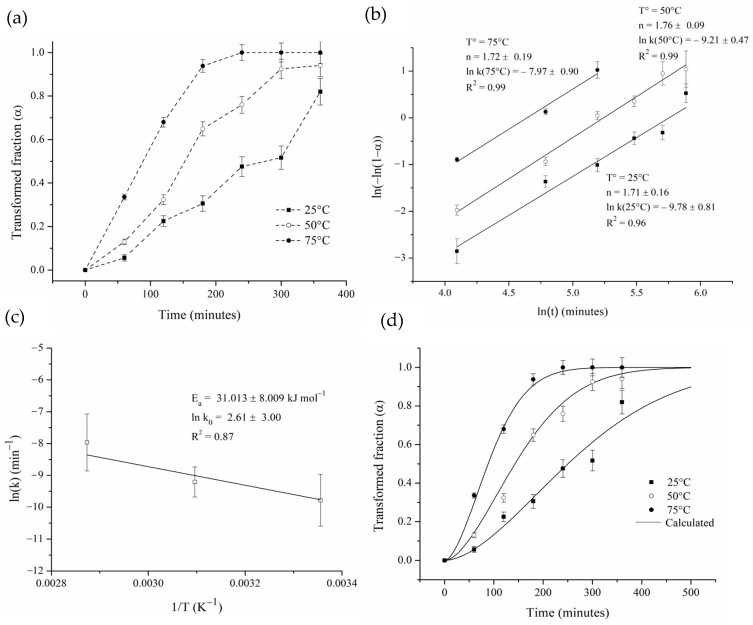
(**a**) Transformed fraction as a function of time for different temperatures, (**b**) linear adjustment using JMAK model, (**c**) Arrhenius plot of ln(k) against 1/T, and (**d**) comparison between experimental and calculated transformed fraction.

To study the oxidation kinetics of the Ag-based solid solution during the reactive hot milling process, the transformed fraction “*α*” was determined using the following equation:

(3) $$\alpha =\frac{∆\alpha_{t}}{∆\alpha_{T}}$$

where “∆*α_t_*” is the variation in the SS-Ag lattice parameter during the hot mechanochemical process in an interval of time “*t*”, and “∆*α_T_*” is the difference between the lattice parameter of the SS-Ag obtained by mechanical alloying and the lattice parameter of pure-Ag. Figure 10a shows the results obtained.

Figure 10b presents the Johnson–Mehl–Avrami–Kolmogorov (JMAK) linear adjustments for the three studied temperatures. A good fit was observed (R^2^ greater than 0.97), indicating that the JMAK model accurately describes the isothermal Zn_2_SnO_4_ formation during the hot mechanochemical process. The kinetics parameters “*n*” and “*k*(*T*)” were calculated from the slopes and intercepts of the plots, respectively. It can be seen that the Avrami exponent “*n*” obtained is in the range of 1.71 to 1.76, which, according to theory, could indicate that the process takes place at a constant nucleation rate and is controlled by the diffusive growth of Zn_2_SnO_4_ in one dimension, or otherwise, that the process involves instantaneous nucleation and is controlled by the diffusive growth of the oxide in three dimensions [45]. In this way, based on the images of cross-sectioned powders after the hot mechanochemical processing (Figure 8b), it is possible to conclude that the oxide growth during the hot milling process is more related to growth in three dimensions than growth in one dimension.

On the other hand, the activation energy “*Ea*” and pre-exponential factor “*k_o_*” were obtained using the Arrhenius equation (Figure 10c). The activation energy “*Ea*” was calculated to be 31.013 ± 8.009 kJ mol^−1^, which is close to the activation energy of oxygen diffusion in Ag reported by Gryaznov [46] (33.9 kJ mol^−1^). Therefore, the kinetics equation for the formation of Zn_2_SnO_4_ from SS-Ag through hot mechanochemical processing under the aforementioned conditions may be written as follows:

(4) $$\alpha \left(t,T\right)=1-e^{-13.58\times e^{\frac{-31013}{R\times T}}\times t^{1.73}}$$

Figure 10d shows the comparison between the simulated curves. The simulated and experimental data fit well, corroborating our claim that the JMAK model accurately describes the process under study. The average relative error found was 6.84%.

Based on the microstructure observed in Figure 8b and the relation between the activation energy “*Ea*” and the Avrami exponent “*n*” obtained from the fits of the experimental data, it is possible to establish that the main mechanism for the oxidation of the Ag-Sn-Zn solid solution during hot mechanochemical processing would be related to a three-dimensional growth of the oxide, limited by diffusion of oxygen after its immediate initial nucleation [45]. This instantaneous nucleation can be understood by considering the high density of the crystalline defects generated in the powders during milling, which can act as preferential sites for oxide formation.

Finally, it is important to highlight that the Ag-Zn_2_SnO_4_ powders synthesized via mechanical alloying and hot mechanochemical processing exhibit microstructural and morphological characteristics that suggest promising applications in the field of photocatalytic materials. Previous studies have demonstrated that Zn_2_SnO_4_ significantly enhances its photoelectric response when forming a heterojunction with Ag [47]. In this context, Huang et al. [48] reported that the improvement in the electrocatalytic activity of Zn_2_SnO_4_ due to Ag incorporation is not only attributed to increased capture of photogenerated electrons, facilitating charge separation, but also to enhanced visible light absorption promoting carrier generation. Additionally, Ag-Zn_2_SnO_4_ powders produced through hot mechanochemical processing show potential in gas-sensing applications. Yan et al. [49] demonstrated that a composite material of Ag-Zn_2_SnO_4_ can detect triethylamine gas at concentrations as low as 1 ppm, with a response time of less than 1 s. These findings suggest that the present study could open new avenues for the development of Zn_2_SnO_4_-Ag photocatalytic and gas-sensing materials with microstructures that can be easily tuned to optimize their performance.

## 4. Conclusions

This work undertook a comprehensive study on the synthesis of Ag-Zn_2_SnO_4_ powders by mechanical alloying and subsequent hot mechanochemical processing. The following main conclusions were obtained:

Regarding the mechanical alloying process, it can be concluded that it is possible to obtain a Ag—6 wt% Sn—6 wt% Zn solid solution with nanoscale crystallite sizes through 60 min of mechanical alloying under the experimental conditions employed. Additionally, a phase transformation sequence was proposed for the formation of the Ag—6 wt% Sn—6 wt% Zn solid solution through mechanical alloying, starting from elemental powders of Ag, Zn, and Sn (reaction (2)).

Concerning the hot mechanochemical processing of Ag-Sn-Zn solid solution powders, it can be concluded that the complete oxidation of the Ag-based solid solution is achievable within 360 min when the process is carried out at 75 °C under the tested conditions. In this regard, the presence of Zn_2_SnO_4_ was detected in the powders subjected to hot mechanochemical processing. FEG-SEM analysis of metallographically prepared powders revealed the presence of nanometric oxide precipitates uniformly distributed within the Ag matrix. These results confirm the effectiveness of the synthesis method in producing powders with a fine and homogeneous distribution of oxides.

Regarding the kinetic study of the hot mechanochemical process, it can be established that mechanochemical oxidation of the Ag-Sn-Zn solid solution follows a JMAK model. Based on the microstructure observed and the relation between the activation energy “*Ea*” and the Avrami exponent “*n*” obtained from the fits of the experimental data, it is possible to establish that the primary mechanism for the oxidation of the Ag-Sn-Zn solid solution during hot mechanochemical processing would be related to a three-dimensional growth of the oxide limited by the diffusion of oxygen after its immediate initial nucleation.

In conclusion, this study can inspire further research and contribute to the development of a new electrical contact material, offering a potential alternative to the environmentally harmful Ag-CdO composites currently used in low-voltage applications. It is recommended that future research focus on the fabrication of electrical contact components from Ag-Zn_2_SnO_4_ powders produced via the proposed method and evaluate their performance under a range of electrical contact conditions. This new consolidated material is expected to exhibit excellent electrical contact properties due to the good adhesion between Zn_2_SnO_4_ and Ag, reducing the probability of oxide surface segregation occurring during operation. Additionally, the nanoscale and homogeneous distribution of Zn_2_SnO_4_ within the Ag matrix would impart optimal mechanical properties to the material for such applications.

## Data Availability

Data are contained within the article.
